# Prolonged Toxicokinetics and Toxicodynamics of Paraquat in Mouse Brain

**DOI:** 10.1289/ehp.9932

**Published:** 2007-07-20

**Authors:** Kavita Prasad, Bozena Winnik, Mona J. Thiruchelvam, Brian Buckley, Oleg Mirochnitchenko, Eric K. Richfield

**Affiliations:** 1 Department of Pathology and Lab Medicine, Robert Wood Johnson Medical School, University of Medicine and Dentistry New Jersey, Piscataway, New Jersey, USA; 2 Environmental and Occupational Health Sciences Institute, Piscataway, New Jersey, USA; 3 Department of Environmental and Occupational Medicine, Robert Wood Johnson Medical School, University of Medicine and Dentistry New Jersey, Piscataway, New Jersey, USA; 4 Rutgers University, Piscataway, New Jersey, USA; 5 Department of Biochemistry, Robert Wood Johnson Medical School, University of Medicine and Dentistry New Jersey, Piscataway, New Jersey, USA

**Keywords:** 20S proteasome, dopamine system, neurotoxicology, oxidative stress, Parkinson disease, proteolytic stress, toxicodynamics, toxicokinetics, ubiquitin-proteasome system

## Abstract

**Background:**

Paraquat (PQ) has been implicated as a risk factor for the Parkinson disease phenotype (PDP) in humans and mice using epidemiologic or experimental approaches. The toxicokinetics (TK) and toxicodynamics (TD) of PQ in the brain are not well understood.

**Objectives:**

The TK and TD of PQ in brain were measured after single or repeated doses.

**Methods:**

Brain regions were analyzed for PQ levels, amount of lipid peroxidation, and functional activity of the 20S proteasome.

**Results:**

Paraquat (10 mg/kg, ip) was found to be persistent in mouse ventral midbrain (VM) with an apparent half-life of approximately 28 days and was cumulative with a linear pattern between one and five doses. PQ was also absorbed orally with a concentration in brain rising linearly after single doses between 10 and 50 mg/kg. The level of tissue lipid peroxides (LPO) was differentially elevated in three regions, being highest in VM, lower in striatum (STR), and least in frontal cortex (FCtx), with the earliest significant elevation detected at 1 day. An elevated level of LPO was still present in VM after 28 days. Despite the cumulative tissue levels of PQ after one, three, and five doses, the level of LPO was not further increased. The activity of the 20S proteasome in the striatum was altered after a single dose and reduced after five doses.

**Conclusions:**

These data have implications for PQ as a risk factor in humans and in rodent models of the PDP.

The Parkinson disease phenotype (PDP) is a complex trait resulting from the interaction of multiple risk factors. The clearest risk factors in humans are genetic with autosomal dominant and recessive forms contributing to familial subtypes, but polymorphisms in other genes may contribute to the sporadic subtype (Cookson et al. 2005; Farrer 2006; Feany 2004; Ross and Farrer 2005). Nongenetic contributions to risk include lifestyle, diet, age, and exposures to xenobiotics (Chen et al. 2005; Firestone et al. 2005; Gorell et al. 2004; Logroscino 2005; Priyadarshi et al. 2001; Tanner 2003). Different classes of compounds including pesticides and metals have been implicated. 1-Methyl 4-phenyl-1,2,3,6-tetrahydropyridine (MPTP) is one xenobiotic producing a form of the PDP that differs from the typical sporadic form in speed of onset, systemic administration, and lack of Lewy body formation (Forno et al. 1993). Paraquat (PQ, 1,1′-dimethyl-4,4′-bipyridinium) is a pesticide, dessicant, and defoliant and may be the only specific xenobiotic implicated in the sporadic human form of the PDP (Dinis-Olivera et al. 2006). Human epidemiologic data implicating PQ include individual case reports and case–control studies (Liou et al. 1997; Sanchez-Ramos et al. 1987). Proving a xenobiotic is a risk factor for the PDP in humans is difficult because of challenges associated with documenting and measuring exposures from conception through diagnosis and the potentially long interval between the exposure and the disease onset.

Paraquat was first produced commercially as a pesticide in 1961 (Pesticide Action Network United Kingdom 2007). Although banned in several countries because of acute toxicity, it remains one of the most widely used pesticides in the world including the United States and remains in use on more than 100 crops. For example, nearly 1 million pounds of PQ were used on an extensive range of crops in 2005 in California alone, reflecting an increase in use from previous years (California Department of Pesticide Regulation 2005). PQ is one of the most common workplace exposure risks in Costa Rica (Partanen et al. 2003) and by drift of aerial applications can affect nearby individuals (Ames et al. 1993). The risk versus benefit for paraquat use is complex. Evaluation must weigh the beneficial effect of PQ on plant productivity versus its yet unproven adverse effect in the human brain. However, evaluating potential brain effects in humans is difficult and must rely initially on animal studies.

Experimental animal data using PQ is easier to obtain than human data and exposure to PQ results in rodent models of the PDP that are compelling. PQ has been demonstrated to preferentially kill dopaminergic neurons in the substantia nigra pars compacta (SNpc) in mice in different laboratories (DiMonte et al. 2001; Dinis-Olivera et al. 2006; Manning-Bog et al. 2003; Thiruchelvam et al. 2000, 2003). It also produces dopamine (DA)-mediated behavioral and biochemical changes in mice and rats (Barlow et al. 2004; Corasaniti et al. 1991; Ossawska et al. 2005; Thiruchelvam et al. 2005). The effects of PQ may occur after as few as two relatively low (10 mg/kg) doses (McCormack et al. 2005). It is active after both injected and oral routes (Widdowson et al. 1996). Although the reason for preferential dopaminergic neuron loss in models is not known, a role for oxidative stress has been strongly suggested based on *in vitro* and *in vivo* evidence (McCormack et al. 2005; Yumino et al. 2002).

In performing toxicokinetics (TK) studies of PQ in different strains of mice, it became clear that previous data from the brain we collected might be incomplete because of too short a sampling interval limited to 12 hr (Barlow et al. 2003). Presently, multiple long-term studies (weeks) were performed and demonstrate that the half-life of PQ in brain is much longer. Additionally, oral ingestion resulted in brain accumulation similar to ip exposure. Because the oxidative consequences of PQ are well established and may contribute adverse effects, lipid peroxidation and 20S proteasome functional activity were also determined. These data suggest that the adverse effects associated with PQ exposure in humans may be more complex than suspected because of the prolonged retention in brain. PQ continues to serve as a useful compound for modeling the PDP in rodents and understanding mechanisms of neurotoxicity.

## Methods

### Treatment and tissue acquisition

C57BL/6J mice were bred, group housed in a climate-and light-controlled (12/12-hr light/dark, light on at 0600) room at the University of Medicine and Dentistry of New Jersey (UMDNJ). Food and water were provided *ad libitum*. Male mice between 8 and 12 weeks of age were used, with a sample size of four for each condition and time point. Animals were treated humanely and with regard for alleviation of suffering, and all procedures were approved by the UMDNJ Institutional Animal Care and Use Committee (IACUC) and were in accordance with the National Institutes of Health *Guide for the Care and Use of Laboratory Animals* (Clark et al. 1996). Paraquat (Sigma Chemical Co., St. Louis, MO) was made fresh in saline for each injection. PQ was either injected by the ip route (10 mg/kg) or administered orally by gavage (10–50 mg/kg). The dose of 10 mg/kg is similar to what we and others have used in adult mice (McCormack et al. 2005). The same and higher oral doses were used because of an expected lower brain PQ level with that route of exposure. When mice were treated for more than one dose, a Monday–Wednesday–Friday (M-W-F) regimen was used. Mice given multiple doses were given the same dose (10 mg/kg) at the approximate same time of day. The dose was always based on the animal’s current weight. Mice were allowed to survive after administration for 1 hr to 28 days. Mice that were perfused were anesthetized with Nembutal at a dose of approximately 75 μg/g (∼ 1.9 mg/25 g mouse). Nembutal was diluted to 5 g/mL in saline and a typical volume of 0.375 mL used. After adequate anesthesia, mice were perfused with cold isotonic saline (20 mL) before sacrifice when the tissue was used for paraquat determination. Mice were sacrificed by cervical dislocation for all other outcome measures. Brains were harvested, dissected on ice to obtain the regions of interest including frontal cortex (FCtx), striatum (STR), and ventral midbrain (VM) containing the substantia nigra (SN), and immediately frozen with dry ice. Tissues were stored at −80ºC until used in assays described below.

### Paraquat determination

Tissue samples (10–15 mg accurately weighed) placed into a 1.5-mL centrifuge tube were mixed with 150 μL of 12% acetic acid and then sonicated for approximately 20 min. The centrifuge tubes were placed in high-pressure microwaveable Teflon extraction vessels (CEM HP500; CEM Corp., Matthews, NC) and heated in a microwave digestion/extraction system (CEM MARS; CEM Corp.) for 30 min at 50% power. The samples were then centrifuged using a 10-kDa filter (Nanosep 10k Omega Pall Trincor; Omega Pall Corp., Exton, PA) and the filtrate transferred to HPLC/autosample vials and stored at −30ºC until analysis. The PQ separation was carried out on ZORBAX RX-C8, 4.6 mm × 15 cm, 5-μm column (Agilent Technologies, Santa Clara, CA) with reverse-phase column guard 4.3 mm × 1 cm. PQ was eluted at flow rate 0.3 mL/min with a retention time of 5 min monitored by both ultraviolet detector and mass spectrometer (Waters 996 photodiode array detector; Waters Corp., Milford, MA). A 50-μL sample was injected. Gradient elution was established with three-solvent system (Table 1): 0.1% formic acid in water (A), 0.1% formic acid in methanol (B), and 0.1% formic acid in acetonitrile (C). Each calibration curve was prepared separately for each experiment and composed of PQ standards (Sigma Chemicals) with a concentration range of 0–12 ng. As a part of each experiment matrix blanks and spikes were prepared. Matrix blanks were prepared by adding 150 μl of the extraction solvent to untreated mouse brain tissue. The linear range was 0–2 ng/μL. Quantification was carried out using an LCQ ion trap mass spectrometer (Finnigan, San Jose, CA) equipped with an electrospray ionization source (ESI) and operated using Xcalibur 1.3 software (ThermoFischer Scientific Waltham, MA). Quantification of PQ was based on integrated peak areas of the *m/z* 186 ion in selective ion monitoring (SIM) mode with isolation width *m/z* 1.

### Lipid hydroperoxide assay

Frozen tissue samples were quickly homogenized in 10 mM Tris–HCl buffer (pH 7.4) with a tissue sample weight to volume of 1:20 and an equal volume of ExtractR (LPO Assay Kit; Calbiochem, San Diego, CA) saturated with deoxygenated methanol was added. After vortexing, 2 vol of cold deoxygenated chloroform was added and after additional vortexing, the mixture was centrifuged at 1,500 × *g* for 5 min at 4ºC. The bottom chloroform layer was collected and used for the lipid peroxides measurements. The assay measures lipid hydroperoxides directly using redox reactions with ferrous ions. The resulting ferric ions were detected using thiocyanate as a chromogen by measuring absorbance at 500 nm. Hydroperoxide values in samples were calculated using the equation obtained from the linear regression of a standard curve and finally adjusted to the protein content in each sample.

### Proteasome activity

Tissue samples were sonicated at a setting of 25 (range 0–100, Branson Sonifier 250 (Branson Ultrasonics, Danbury, CT) for 10 sec in 50 mM Hepes, pH 7.5, 150 mM NaCl, 5 mM EDTA, and 1% Triton X-100 in the presence of protease inhibitors (leupeptin 0.5 μg/mL, aprotinin 1.0 μg/mL, pepstatin A 0.7 μg/mL, and antipain 50 μg/mL) at a weight to volume of 20:1. Samples were spun at 14,000 rpm for 15 min at 4ºC with removal of the supernatant to a new tube. The protein concentrations were determined and samples diluted to 3 μg/μL before use. Black flat-bottom microtiter plates were filled in order with incubation buffer (50 mM Tris–HCl, pH 7.5; tissue supernatant, 5 μL), and substrates (5 μL) to a final volume of 200 μL on ice. Samples were run in duplicate or triplicate. Control tissue in the absence or presence of the inhibitor MG-132 (100 μM) was also analyzed. Plates were analyzed using a Tecan Genios (Tecan US, Durham, NC) plate reader at 37ºC using an excitation filter (360 nm), an emission filter (465 nm), a manual gain of 60, 3 flashes per reading, with measurements performed every 2 min for a total of 60 min with shaking between each reading. Suc-Leu-Leu-Val-Tyr-AMC (20 μM, Boston Biochem, Boston, MA) was used to determine the chymotrypsin-like (ChymT-like) activity and Z-Leu-Leu-Glu-AMC (80 μM; Boston Biochem) was used to determine peptidylglutamyl peptide hydrolyzing (PGPH) activity. An increasing linear curve was observed after a short delay for all tissue samples for the 60-min duration. Specific activity was determined by subtracting activity from wells containing MG-132 (100 μM). Protein concentrations were determined once more after dilutions, and those values used for the final calculation of activity in units of relative fluorescence units (RFU) per microgram protein per hour.

### Statistical analysis

Levels of PQ in toxicokinetic studies, concentration of lipid peroxides, and proteasomal activity were analyzed using two-factor analysis of variances (ANOVAs) with either treatment and time point or treatment and number of doses as between group factors. Post hoc assessments were carried out based on main effects or interactions as appropriate. For all analyses, *p*-values of ≤ 0.05 were considered statistically significant.

## Results

**Figure 1:**
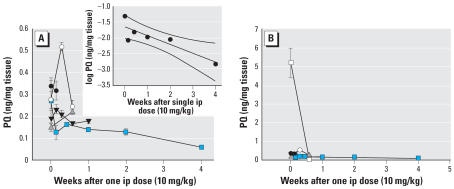
Time course of PQ in ventral midbrain (VM) and liver. (*A*) Brain values for PQ. (*B*) Brain and liver values for PQ. PQ (10 mg/kg) was injected by the ip route and tissue harvested at different time points. A total of five independent time–course studies were performed with each time point from each study having sample size of four as indicated by a mean and SE for a total of 72 mice. Each type of symbol represents an independent experiment. Values in the VM (*A*) had greater variability at early time points (1 hr–3 days) compared with later time points (4 day–4 weeks). A plot of the log PQ concentration versus time (using the data from the experiment with the longest time point, filled squares) suggests a roughly linear decrease (*r*^2^ = 0.76, linear regression with 95% confidence interval) with a brain half-life of about 4 weeks (*A*). At no time point was PQ entirely eliminated from brain in contrast to liver (open square) where elimination was complete at 4 days (*B*; all other symbols represent VM from *A*). Levels in the liver (and other organs) are much higher than those in brain.

The ventral midbrain (VM) was used as the primary region for PQ measurement because it is the primary region with cell loss in Parkinson disease and previous data from our laboratory demonstrated that other brain regions (striatum, frontal cortex, and cerebellum) had similar levels of PQ 12 hr after administration (Barlow et al. 2003). In previous TK studies, radioactive PQ was used, whereas a safer and more sensitive liquid chromatography–mass spectrometry assay has been developed and used in this study. Clarifying the TK we performed five independent time-course studies of differing duration in the C57BL/6J strain (Figure 1). These studies support existing data indicating only a fraction of the systemic dose enters into the brain compared with the liver (Figure 1B) (Barlow et al. 2003). PQ was persistent in brain with failure to demonstrate complete elimination even after 4 weeks (Figure 1A). There was greater variation in PQ concentrations at early time points (< 3 days) compared with later time points (> 3 days). When the longest time course was plotted using a log scale, the elimination appeared linear, with a brain half-life of approximately 4 weeks (*r*^2^ = 0.76). The PQ detected throughout the time course was unchanged in structure, with no breakdown products being detected. It is unclear whether the slow decline in brain was due to metabolism of PQ to an undetected metabolite or the very slow elimination of native PQ.

**Figure 2:**
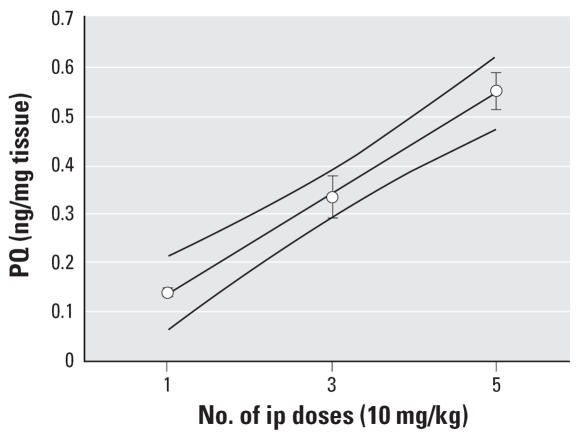
Effect of repeated doses of PQ. PQ (10 mg/kg) was administered ip for a total of one, three (M-W-F), or five (M-W-F-M-W) doses and measured one week after the last dose in the VM. There was a linear increase in the amount of PQ (*r*^2^ = 0.99) with the number of doses (linear fit and 95% confidence interval). Each value represents a mean ± SE from four mice.

**Figure 3:**
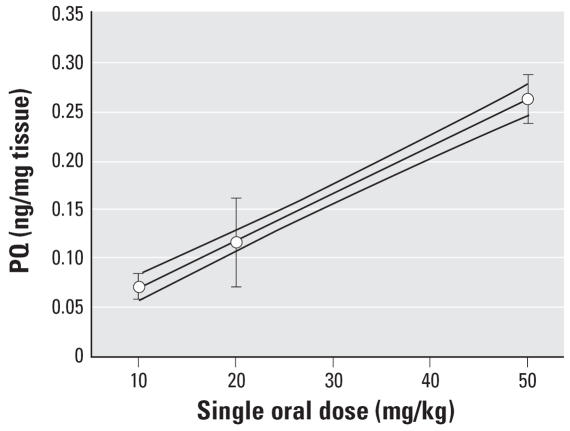
Effect of oral administration of PQ. PQ was administered once at each of three doses (10, 20, and 50 mg/kg po) and assayed after 4 and 6 hr. As similar values were found at both time points only the 6-hr data are presented. There was a linear relationship between dose and level in the VM (*r*^2^ = 1.0, 95% confidence intervals) using the 6-hr time point. Each value represents a mean ± SE from four mice.

We then sought to understand the biological effects of repeated doses of PQ in brain. The same dose (10 mg/kg) was administered ip 3 times/week (M-W-F) for a total of one, three, or five doses (Figure 2). There was a linear increase in PQ in the VM (*r*^2^ = 0.99). When administered orally (10, 20, or 50 mg/kg) a similar linear increase in PQ levels was observed in the VM following an increasing dose (*r*^2^ = 1.00; Figure 3).

**Figure 4:**
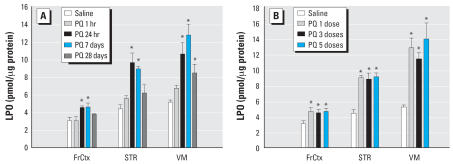
Levels of lipid peroxidation (LPO) in three brain regions after PQ exposure. The level of LPO was measured in VM, striatum (STR), and frontal cortex (FCtx) after different durations of exposure to (*A*) a single dose of PQ (10 mg/kg) or (*B*) 1 week after a total of one, three (M-W-F), or five (M-W-F-M-W) doses of PQ (10 mg/kg). Greatest effects were identified in the VM, lesser in STR, and least in FCtx, as previously reported for a single 24-hr time point. Significant effects were present at 24 hr and 7 days in all regions but were still present up to 28 days in VM. The level of LPO was not significantly increased between one and five doses of PQ. However, each region demonstrated significantly elevated LPO with VM > STR > FCtx. Each value represents a mean ± SE from four mice. (*A*) *Significantly different from saline for respective region, all post hoc *p* < 0.005. (*B*) *Significantly different from saline for respective region, all post hoc *p* < 0.026).

Treating a separate group of mice using similar time points and doses, we measured the tissue level of lipid peroxides (LPO) and the functional activity of the 20S proteasome as toxicodynamic (TD) responses to PQ. We measured LPO levels in three regions. The level of LPO after a single ip dose (10 mg/kg) was increased in as short a time as 1 hr (although not significantly) and persisted for up to 28 days. The overall ANOVA for a single dose measured in three regions at four time points was significant (*F*_4,15_ = 24.9 *p* < 0.0001) with a significant effect for region (*F*_2,15_ = 61.0 *p* < 0.0001) and an interaction of region with time point (*F*_8,30_ = 3.0, *p* = 0.013). (Figure 4A). Maximal effects occurred between 1 and 7 days. Effects were highest in VM, lower in the STR, and least in FCtx. Interestingly, repeated dosing did not result in an increase in the amount of LPO (Figure 4B). Multiple doses of PQ significantly increased the level of LPO in each region (region ANOVAs: VM, *F*_3,12_ = 9.9, *p* = 0.001; STR, *F*_3,12_ =17.9, *p* < 0.0001; FCtx, *F*_3,12_ = 3.8, *p* = 0.04) when measured after 1 week compared with saline.

**Figure 5:**
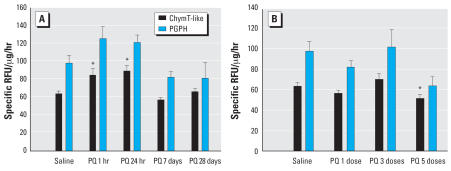
Functional proteasome activity in STR following PQ. The activity of the 20S proteasome was measured in the STR after different durations of exposure to a single dose of PQ (10 mg/kg) (*A*) or 1 week after a total of one, three (M-W-F), or five (M-W-F-M-W) doses of PQ (10 mg/kg) (*B*) in mice also analyzed for LPO levels using other tissue samples (Figure 4). An assay for the chymotrypsin-like activity (ChymT-like) and the peptidylglutamyl peptide hydrolyzing activity (PGPH) of the 20S proteasome were run. Each value represents a mean ± SE from four mice. *Significantly different from saline with all post hoc *p* < 0.03.

The ChymT-like and PGPH activities of the 20S proteasome were measured using the remaining piece of striatal tissue and were acutely elevated at 1 and 24 hr after a single ip dose of PQ in the STR (Figure 5A). This effect was not present 7 or 28 days after a single dose. There was a significant overall effect of PQ on ChymT-like activity (*F*_4,22_ = 9.6, *p* < 0.001) compared with saline with a significant increase in activity seen after 1 and 24 hr (post hoc *p*-values < 0.003). There was a similar significant overall effect of PQ on PGPH activity (*F*_4,22_ = 3.9, *p* = 0.016) compared with saline, but the post hoc tests did not reveal a significant increase after 1 and 24 hr, although a trend was present (post hoc *p*-values < 0.12). The variances were greater for the PGPH activity compared with the ChymT-like activity and sample sizes were quite small (*n* = 4). Multiple doses of PQ resulted in a significant overall effect of PQ on ChymT-like (*F*_3,20_ = 4.1, *p* < 0.02) activity compared with saline, with a significant decrease in activity seen after five doses (post hoc *p* = 0.029). Multiple doses of PQ did not result in a significant overall effect of PQ on PGPH activity (*F*_3,19_ = 3.4, *p* = 0.07) compared with saline although there was a trend with the direction and magnitude of change similar to those seen with ChymT-like activity. Repeated doses resulted in no significant effects after one or three doses, but five doses significantly decreased ChymT-like activity 7 days after treatment (Figure 5B).

## Discussion

This study demonstrates that PQ persists in the VM of mice for a prolonged time, with a half-life of approximately one month. The reason for this persistence in brain in contrast to that in other organs is not known, but this persistence may contribute to its prolonged adverse effects. PQ elimination in laboratory animals and humans from blood and organs other than the brain has always been reported in hours and days (Dey et al. 1990; Houze et al. 1990). It is unclear if the slow loss of PQ from the VM was due to metabolism, export, or both. The TK of PQ in brain has not been well studied due to its low concentration and technical demands of the available assays. The long half-life of the charged PQ was surprising. Our previous report using [^14^C]-PQ failed to demonstrate complete excretion but was limited to a 12-hr duration (Barlow et al. 2003).

Additional data supported this prolonged half-life. PQ was linearly cumulative in VM between one, three, and five doses, supporting the lack of elimination between doses. Given a half-life of approximately 28 days in mouse brain, the accumulation of PQ with repeated exposure would be expected. If this TK pattern applies to humans, repeated exposures may be more adverse than suspected and the limits to exposure may need to be reconsidered. Given the prolonged duration of PQ in brain, adverse effects should be evaluated in terms of cumulative brain concentration and duration of exposure rather than dose, number of doses, or interval between doses.

Other studies using radiolabeled PQ found that it was absorbed by all exposure routes tested, including intravenous, intragastric, dermal, and pulmonary (Chui et al. 1988; Dey et al. 1990). PQ in brain after oral dosing was previously demonstrated in rat and the concentration was higher after 10 doses compared with 1, supporting its accumulation (Widdowson et al. 1996). Thus, oral, dermal, and inhalational exposures to PQ are all feasible as in models and relevant to human exposure. Given this long half-life and accumulation of PQ in mouse brain and the potential for all routes of exposure to contribute, this information should be considered in evaluating the human risk associated with PQ.

Despite low brain PQ concentrations, adverse effects *in vivo* are clearly based on multiple measures performed by us and others including the loss of DA neurons, alterations in DA level and metabolism in the striatum, and changes in oxidative status using different species of rodents (Di Monte et al. 2001; Fredriksson et al. 1993; Gonzalez-Polo et al. 2004; Hara et al. 1991; Manning-Bog et al. 2002, 2003; McCormack et al. 2002, 2005; Miranda-Contreras et al. 2005; Ossawska et al. 2005; Shimizu et al. 2003; Thiruchelvam et al. 2000a, 2000b, 2002, 2003, 2005). This article demonstrated prolonged elevated oxidative stress after a single exposure, with brain PQ levels between 0.1 and 0.2 ng/mg tissue. Based on published work by others we can extrapolate that brain concentrations near 0.3 ng/mg tissue (following a minimum of two 10-mg/kg doses) result in neuronal death in the SN of mice (McCormack et al. 2005). Although the mechanism(s) for the adverse effects of PQ on dopaminergic neurons is incompletely understood, oxidative stress (a well-known consequence of PQ) or other processes are likely to play a role (Andersen 2004).

Our data on the level of lipid peroxidation confirmed significant elevation of reactive oxygen species (ROS) after single and repeated doses of PQ (McCormack et al. 2005; Thiruchelvam et al. 2005; Yang and Sun 1998; Yang and Tiffany-Castiglioni 2005; Yumino et al. 2002). The lack of difference between one, three, and five doses of PQ on LPO levels might suggest a ceiling effect due to a limited number of targets or a tissue compensatory response to the induced stress over time. We expect that ROS formed would cause irreversible oxidative damage to proteins. Moderately carbonylated proteins undergo degradation by the ubiquitin/proteasome pathway (UPS) (Poppek and Grune 2006), which might explain the temporal activation of proteasomal activity at 1 and 24 hr in striatum after a single dose of PQ. Sublethal proteasome inhibition induces neurons to increase proteasome activity and promotes resistance to oxidative injury (Lee et al. 2004), whereas oxidative stress can increase proteasome activity early in the sequence leading to cell death *in vitro* (Holtz 2006). Interestingly, PQ has been shown to produce intracellular protein aggregation in neurons after repeated dosing, although the mechanism is unknown (Manning-Bog et al. 2002). Excessive generation of oxidative stress alone may damage proteasomes directly (Farout 2006) and certain pesticides are known to directly inhibit proteasomal activity (Wang et al. 2006).

The TK and TD of PQ in brain are due to the interaction of genetic and environmental factors. Environmental factors include diet, nutritional status, exposure to other xenobiotics, age, and potentially many other factors. Given that PQ may not be metabolized in brain and the intact molecule may be solely responsible for adverse effects, the TK in brain may be related to a limited number of proteins participating in the bidirectional transport into and out of the brain and cells. Transporters thought to contribute include the amino acid transporters for crossing the blood–brain barrier (McCormack and Di Monte 2003; Shimizu and Ohtaki. 2001). Transporters involved in cellular and subcellular transport have not been clearly established but may include organic cation transporters (Jack et al. 2000; Smith 1982; Sokol et al. 1987; Wu et al. 1998). The use of different inbred or recombinant inbred mouse strains will be useful for identifying the mechanisms and genes participating in the TK and TD of PQ. Those mechanisms and genes may then be examined in relationship to PQ vulnerability in humans.

## Conclusions

The TK and TD of paraquat are complex. The accumulation and persistence of PQ in brain suggest the human exposure limits to PQ might need to be reconsidered. Models of the PDP using PQ should be evaluated in terms of total brain accumulation and duration of exposure rather than number and frequency of doses.

## Figures and Tables

**Table 1:** Gradient elution for three solvent systems for HPLC.

		Solvent system (%)		
Time (min)	Flow (mL/min)	A	B	C
0.00	0.3	60	25	15
5.00	0.3	50	25	25
6.00	0.3	30	25	45
15.00	0.3	30	25	45
16.00	0.3	60	25	15
20.00	0.3	60	25	15
